# Value of ultrasonography in determining the nature of pleural effusion: Analysis of 582 cases

**DOI:** 10.1097/MD.0000000000030119

**Published:** 2022-08-19

**Authors:** Ting Wang, Ge Du, Liping Fang, Yang Bai, Zishuang Liu, Li Wang

**Affiliations:** a Department of Respiratory Medicine, Xi’an People’s Hospital (Xi’an No. 4 Hospital), Xi’an 710004, China; b Department of Rehabilitation Center for Elderly, Beijing Rehabilitation Hospital Affiliated to Capital Medical University, Beijing 100144, China; c Department of Medical Ultrasonics, Xi’an People’s Hospital (Xi’an No. 4 Hospital), Xi’an 710004, China; d Department of Radiology, Xi’an People’s Hospital (Xi’an No. 4 Hospital), Xi’an 710004, China.

**Keywords:** echogenicity, exudate, septation, transudate, ultrasonography

## Abstract

To explore the value of ultrasonography in the auxiliary diagnosis of pleural effusion, we retrospectively analyzed the ultrasonographic findings of 275 exudates and 307 transudates and summarized the ultrasonographic image features of pleural effusion according to patients’ primary diseases. The findings of thoracic ultrasonography performed before the initial thoracentesis in 582 patients with subsequently confirmed exudative/transudative pleural effusion were analyzed with regard to the sonographic features of pleural effusion. In 275 cases with exudates, thoracic ultrasonography showed a complex septate appearance in 19 cases (6.9%), complex nonseptate appearance in 100 cases (36.4%), complex homogenous sign in 46 cases (16.7%), and pleural thickness > 3 mm in 105 cases. In contrast, in 307 patients with transudates, most patients (97.1%) had bilateral pleural effusion. Ultrasonographic images displayed anechoic appearance and absence of pleural thickening in a vast majority of cases (306, 99.7%; 301, 98%). These positive findings in the exudate were statistically higher than those in their counterparts (*P* < .05). In the empyema subgroup, the proportion of complex septate appearance, complex nonseptate appearance, complex homogenous sign, and pleural thickening was the highest, at 19/41, 12/41, 10/41, and 30/41, respectively. Ultrasonography is valuable in defining the nature of pleural effusion. Some sonographic features of pleural effusion, such as echogenicity, septation, and pleural thickening, may indicate a high risk of exudative pleural effusion.

## 1. Introduction

The pleural cavity is a potential space between the visceral and parietal pleurae. The outer parietal pleura is attached to the chest wall, and the inner pleura (visceral pleura) covers the lungs, via the blood vessels, adjoining structures, bronchi, and nerves. The pleural cavity is a thin space between these 2 pleural layers.^[1]^ Normally, there is a thin layer of fluid (5–15 mL) between the 2 serous membranes, which acts as a lubricant during respiratory movements. Fluid accumulation occurs through various mechanisms, including increased pulmonary capillary pressure, enhanced pleural membrane permeability, decreased oncotic pressure, and lymphatic flow obstruction.^[2–4]^

Based on its pathogenesis, pleural effusion can be divided into exudative pleural effusion (EPE) and transudative pleural effusion (TPE). The former is mostly caused by diseased pleural surfaces, such as pleural tuberculosis and pleural carcinomatosis.^[5,6]^ The latter is caused by systemic factors, such as congestive heart failure and liver cirrhosis, which influence the absorption and formation of pleural fluid.^[7]^ Clinical diagnosis relies on biochemical examination of pleural effusion obtained by thoracentesis.^[8]^ However, this examination cannot be performed in elderly patients who have poor condition or are bedridden because of its invasiveness.^[9]^

Ultrasonography is characterized by high sensitivity and accuracy in identifying and localizing pleural effusion. It has been widely used in the localization and quantification of pleural effusion because of its simplicity, safety, and high acceptance by patients.^[10]^ Limited data are available regarding the accuracy of specific thoracic ultrasonography (TUS) findings in the diagnosis of pleural effusion. This study aimed to evaluate the value of ultrasonography in differentiating EPF from TPF by comparing imaging characteristics. To the best of our knowledge, this is the first study to summarize the ultrasound imaging features of pleural effusion in different primary diseases.

## 2. Materials and Methods

### 2.1. Subjects

We recruited 582 patients with pleural effusion diagnosed by ultrasound imaging admitted to the department of our hospital between January 2016 and December 2020. The ultrasonographic imaging characteristics of 582 patients before treatment were retrospectively analyzed. After ultrasound examination, all patients underwent thoracentesis. The nature of pleural effusion was defined according to Light criteria.^[11]^ Pleural exudates met at least one of the following criteria, whereas pleural transudates met none: (1) pleural effusion/serum protein ratio > 0.5, (2) pleural effusion/serum lactate dehydrogenase (LDH) ratios > 0.6, (3) pleural effusion LDH level was higher than 2/3 of the upper limit of the normal serum value. Eventually, 275 patients with exudates and 307 patients with transudates were identified. General information, including age, sex, and smoking history, was collected from inpatient medical records.

This study was approved by the Institutional Committee for Research involving human subjects at Xi’an People’s Hospital (Xi’an No. 4 Hospital). All participants were informed of the purpose of the study, and written informed consent was obtained from them.

### 2.2. Ultrasound instrument

A Mindray M9 ultrasonoscope with convex and linear array probes was used with a probe frequency of 3.5 MHz.

### 2.3. Ultrasonography

**Figure 1.**
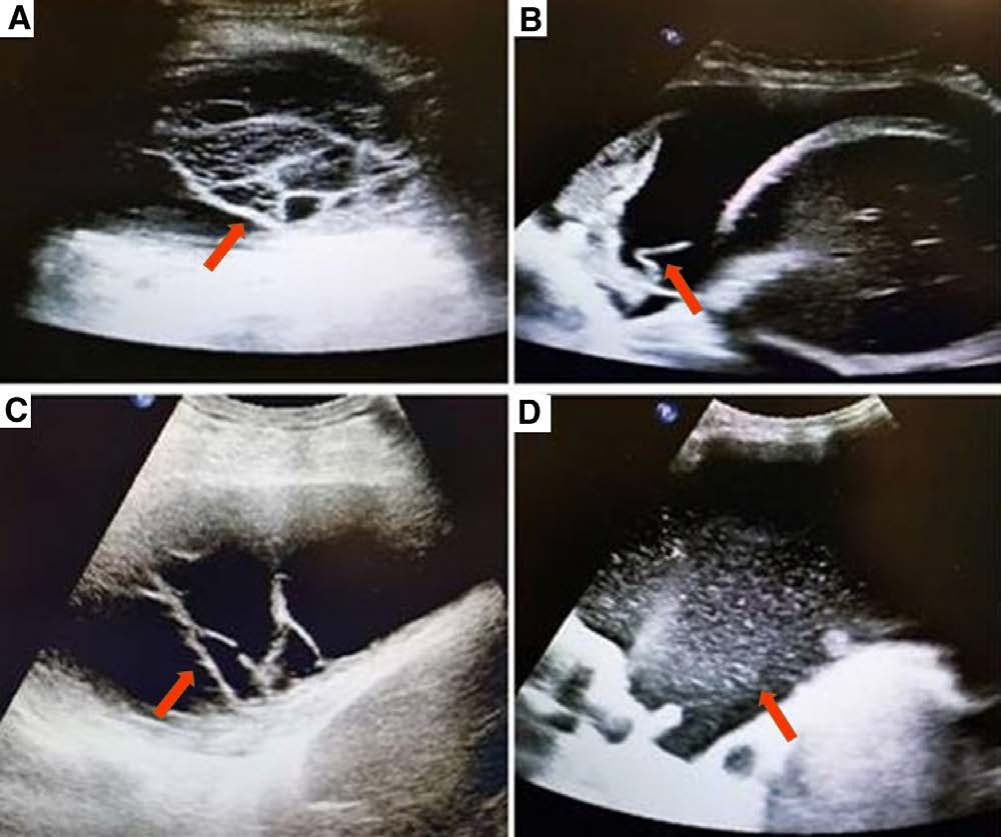
(A) A 44-year-old man with right empyema. TUS showed complex septate appearance and septa were found inside the effusion, as indicated by the red arrow. (B) A 28-year-old woman with left complicated parapneumonic pleural effusion had complex nonseptate appearance in ultrasonographic image. Echogenic material could be seen, as shown by the red arrow, presenting heterogeneously in anechoic space. (C) A 56-year-old woman who was diagnosed as systemic lupus erythematosus (SLE) with left pleural effusion and had complex nonseptate appearance in ultrasonographic image. As indicated by the red arrow, heterogeneous echogenic material could be observed. (D) A 46-year-old man with left empyema. Complex homogenous fluid could be found by the TUS and a large number of light spots could be noted, shown by the red arrow.

The patient was placed in the sitting position with the back facing the examinee. His or her upper body lies on the back of the chair, with hands holding his or her head to move the scapula upward and widen the intercostal space. The ultrasound probe detected the 7th rib to 8th rib of the posterior axillary line and provided a cross-sectional observation. TUS was performed before pleural tap by 4 operators, YB, WFC, BRH, QZ, and 2 investigators (YB, WFC) who had no clinical information concerning the patients retrospectively analyzed the images. The TUS image characteristics were defined according to the previously published criteria, as shown in Figure 1.^[12]^ Effusion is considered anechoic if it is echo-free, complex septate if septa or fibrin strands are found inside the effusion, and complex nonseptate if echogenic material is present heterogeneously in the anechoic space. Additionally, complex homogeneity is defined as the presence of echogenic space homogeneously throughout the pleural space.

Clinical diagnosis was made by a clinician relying on biochemical examination of pleural effusion and other relevant supplementary examinations.

### 2.4. Statistical analysis

We used Microsoft Excel for data collection, and statistical analysis was performed using the SPSS 26.0 statistical package. The count data were expressed as numbers and percentages (%), and the chi-square test was used to compare variables between the 2 groups. If the value of expected cases in 1 cell was ≥1 but <5, we adopted a continuity-adjusted formula for the chi-square test. Fisher exact test was used if a cell had few expected cases (ie, <1) in the table. Statistical significance was set at a *P* value < 0.05.

## 3. Results

In the abovementioned 5-year period, 582 patients with confirmed primary diseases were obtained by retrospective analysis, as shown in Table 1. Of 275 patients with exudative pleural effusion, there were 152 men and 123 women with an age range of 17 to 83 years. TUS revealed that 214 patients (77.8%) had unilateral pleural effusion. In 307 patients with transudates, 148 men and 159 women aged 19 to 84 years were studied. Unilateral pleural effusion was noted in 9 patients, accounting for only 2.9%, and the difference was statistically significant (*P* < .05). Moreover, in the group of patients with exudates, a great majority of cases accepted treatment for the first time, accounting for 76.0%, while, in the other group, only 18.9% of patients were newly treated (*P* < .05). In patients with exudates, the main primary diseases were complicated parapneumonic pleural effusion (CPPE, 24.7%), empyema (14.9%), tuberculous effusion (28.0%), malignant pleural effusion (MPE, 25.1%), and rheumatic disease (4%). The primary diseases in patients with transudates were heart failure (71.0%), liver cirrhosis (16.9%), and nephrotic syndrome (12.1%).

**Table 1:** Baseline demographic and clinical characteristics.

Characteristics	Exudates (n = 275)		Transudates (n = 307)		*P* value
	No.	%	No.	%	
Gender					>0.05
Male	152	55.3	148	48.2	
Female	123	44.7	159	51.8	
Age (yr)					>0.05
<60	109	39.6	126	41.0	
≥60	166	60.4	181	59.0	
Initial treatment					<0.05*
Yes	209	76.0	58	18.9	
No	66	24.0	249	81.1	
Location of PE					<0.05*
Unilateral	214	77.8	9	2.9	
Bilateral	61	22.2	298	97.1	
Primary disease					
CPPE	68	24.7	0	0	
Empyema	41	14.9	0	0	
Pleural tuberculosis	77	28.0	0	0	
MPE	69	25.1	0	0	
Rheumatic diseases	11	4.0	0	0	
Heart failure	9	3.3	218	71.0	
Cirrhosis	0	0	52	16.9	
Nephrotic syndrome	0	0	37	12.1	

CPPE = Complicated parapneumonic effusion, MPE = Malignant pleural effusion, PE = Pleural effusion.

*means the difference was statistically significant.

Comparing the ultrasonographic imaging characteristics of the 2 groups, as shown in Table 2, there were differences in the presence or absence of echogenicity, loculations, and pleural thickening. Specifically, in 275 patients with exudates, TUS showed complex septate appearance in 19 patients, complex nonseptate appearance in 100 patients, and complex homogenous sign in 46 patients, accounting for 6.9%, 36.4%, and 16.7%, respectively, and pleural thickness >3 mm was noted in 38.2% of patients. Ultrasonographic images displayed anechoic appearance and absence of pleural thickening in the vast majority of patients with transudate (306, 99.7%; 301, 98%), and the difference was statistically significant (*P* < .01).

**Table 2:** Characteristics of the ultrasonograph in 2 groups.

Characteristics	Exudates (n = 275, %)	Transudates (n = 307, %)	*P* value
TUS images characteristics			0.000*
Anechoic	110, 40	306, 99.7	
Complex septated	19, 6.9	0, 0.0	
Complex nonseptated	100, 36.4	0, 0.0	
Complex homogenous	46, 16.7	1, 0.3	
Pleural thickness			0.000*
≤3 mm	170, 61.8	301, 98.0	
>3 mm	105, 38.2	6, 2.0	

TUS = thoracic ultrasonography.

**Figure 2.**
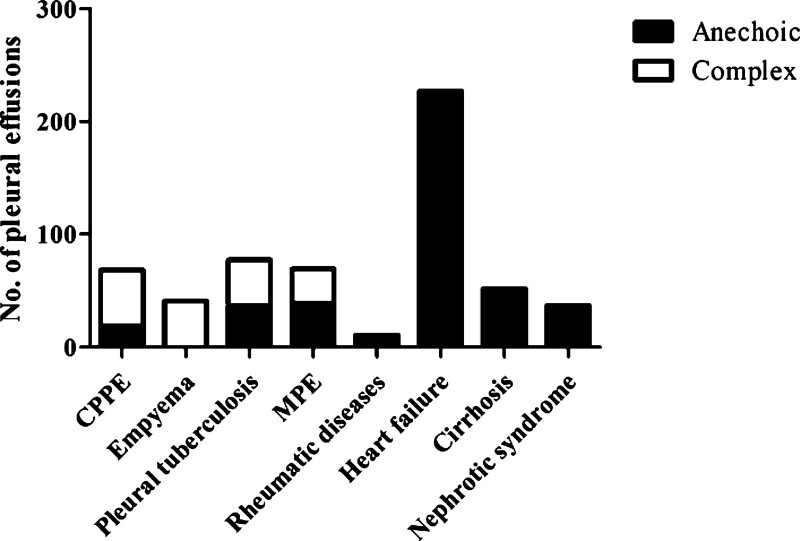
TUS findings of anechoic vs complex with various etiologies of the diseases.

The ultrasound findings of the different diseases are listed in Table 3. In the subpopulations of empyema, the proportion of complex septate appearance, complex nonseptate appearance, complex homogenous sign, and pleural thickening was the highest at 19/41,12/41, 10/41, and 30/41, respectively. In contrast, in the subgroups of patients with liver cirrhosis and nephrotic syndrome with transudate, no patient exhibited the abovementioned ultrasonographic image appearance. Figure 2 displays the relationship between the anechoic and complex TUS findings with various original diseases.

**Table 3:** Ultrasonographic features of different primary diseases in patients with exudates.

	Anechoic	Complex septated	Complex nonseptated	Complex homogenous	Pleural thickness (≤3 mm)	Pleural thickness (>3 mm)
CPPE	19	0	41	8	41	27
Empyema	0	19	12	10	11	30
Pleural tuberculosis	37	0	29	11	40	37
MPE	39	0	16	14	58	11
Rheumatic diseases	7	0	2	2	11	0
Heart failure	226	0	0	1	221	6
Cirrhosis	52	0	0	0	52	0
Nephrotic syndrome	37	0	0	0	37	0

CPPE = complicated parapneumonic effusion, MPE = malignant pleural effusion.

## 4. Discussion

In our study on 582 patients with pleural effusion that evaluated the diagnostic accuracy of TUS for distinguishing transudates from exudates, we found that some sonographic features of pleural effusion, such as echogenicity, septation, and pleural thickening, had a high frequency of appearance in exudative pleural effusion. Furthermore, the frequency of these characteristics was higher in patients diagnosed with empyema.

Although some scholars have shown interest, there is still a paucity of data evaluating the accuracy of TUS in determining the nature of pleural effusion. In 1992, Yang et al first used high-frequency, real-time sonography to determine the nature of pleural effusion.^[13]^ Previously, most reported studies were performed using contact B-scans.^[14–16]^ Yang et al analyzed sonographic images of 320 patients with pleural effusion. Their results showed that all patients with transudates presented anechoic appearance on ultrasonography (96/96), whereas anechoic effusion could be a TPE or EPE (33.9%). Moreover, internal echogenicity of complex patterns and pleural thickening tended to occur in EPE. Their conclusion was consistent with ours, while the proportion of exudates with a complex septate appearance was higher, and the ratio of complex nonseptate signs in exudates was lower in their study. It is possible that the primary diseases in our cohort were CPPE and pleural tuberculosis, unlike MPE, which was dominant in their study. Patients have a relatively high possibility of receiving treatment at the early stage of the disease. This prevents fibrin deposition and production of fibrous septation in pleural effusion with progression of the primary disease.^[17]^ In another study, ultrasonography was considered an alternative to aspiration in determining the nature of pleural effusion, especially in older individuals.^[18–20]^ In their results, anechoic effusion was observed in 100% transudates and 14% exudates. However, another study published in the previous year examined the TUS findings of 300 consecutive pleural effusions and found that an anechoic appearance was associated with 56% of exudative effusions compared to 44% of transudative effusions. Complex-appearing effusion, which had a positive value of 90%, was a high predictor of exudation effusion.^[12]^ These contradictory results could be attributed to the different stages of some primary diseases in various studies. For example, parapneumonic pleural effusion (PPE) can be divided into uncomplicated parapneumonic pleural effusion (UPPE), CPPE, and empyema depending on the disease course.^[21,22]^ Fibrin deposition and fibrous septation, which have an echogenic performance in ultrasound images, are more common in CPPE and empyema than in UPPE.

Furthermore, we summarized the characteristics of ultrasonographic images of pleural effusion caused by different diseases. It is interesting that 9 patients diagnosed with heart failure had EPE, 1 had complex homogenous sign, and 6 had pleural thickening in the ultrasonographic image. This may be due to the fact that patients with heart failure are at risk of pulmonary congestion and infections, which leads to the involvement of infectious factors in the formation of pleural effusion.

In view of the fact that this study was a retrospective investigation that was not integrated with other diagnostic tools to evaluate pleural diseases, the authors are aware of its limitations. In the future, we will design a prospective study that combines ultrasound findings with other examinations, such as chest CT, make comparisons, and evaluate their diagnostic value. We also hope that ultrasound findings could be developed to guide treatment decisions and prognostication in undefined pleural effusions.

## 5. Conclusion

Our analysis of 4 TUS pleural imaging findings in a relatively large cohort (582 cases) found that some sonographic features of pleural effusion, such as echogenicity, septation, and pleural thickening, may be more suggestive of exudates. Among the common diseases leading to exudates, empyema has the highest proportion of positive findings on ultrasonographic imaging.

## Author contributions

All Authors read and approved the manuscript. Detailed contributions are listed as follows: Ting Wang performed the literature review and drafted the manuscript. Ge Du critically revised the manuscript; Liping Fang, Yang Bai collected patients’ data; Zishuang Liu interpreted the data; Li Wang was responsible for the publication fee and overall supervision of the manuscript.
